# Karyotype abnormalities and their clinical significance in a group of chronic myeloid leukemia patients treated with hematopoietic stem cell transplantation

**DOI:** 10.1590/S1516-31802007000400011

**Published:** 2007-07-01

**Authors:** Luize Otero, Maria Helena Ornellas, Alexandre Mello de Azevedo, Rita de Cássia Tavares, Virgínia Pires, Eliana Abdelhay, Luis Fernando Bouzas, Teresa de Souza Fernandez

**Keywords:** Hematopoietic stem cell transplantation, Chronic myeloid leukemia, Chromosome aberrations, Philadelphia chromosome, Prognosis, Transplante de células-tronco hematopoéticas, Leucemia mielóide crônica, Aberrações cromossômicas, Cromossomo Filadélfia, Prognóstico

## Abstract

**CONTEXT AND OBJECTIVE:**

Following hematopoietic stem cell transplantation (HSCT), karyotyping is a valuable tool for monitoring engraftment and disease status. Few studies have examined the prognostic significance of karyotypes in patients who underwent HSCT for chronic myeloid leukemia (CML). The objective of this study was to evaluate the significance of pretransplantation cytogenetic status in relation to outcomes following HSCT in CML patients.

**DESIGN AND SETTING:**

Case series study at Instituto Nacional do Câncer (INCA), Rio de Janeiro, Brazil.

**METHODS:**

Cytogenetic analysis was performed by G banding on 39 patients treated with HSCT.

**RESULTS:**

Thirty-one patients were in the chronic phase and eight were in the accelerated phase. Prior to HSCT, additional chromosomal abnormalities on the Philadelphia (Ph) chromosome were found in 11 patients. The most frequent additional abnormality was a double Ph, which was observed in four cases. Following HSCT, full chimeras were observed in 31 patients (79.5%). Among these, 23 (82.3%) had presented Ph as the sole abnormality. Mixed chimeras were observed in seven patients, of which three had additional abnormalities. Only one case did not present any cytogenetic response. Five patients presented cytogenetic relapse associated with clinical relapse following HSCT. Twenty-seven patients are still alive and present complete hematological and cytogenetic remission.

**CONCLUSION:**

In our study, the presence of additional abnormalities was not associated with worse outcome and relapse risk. Also, no differences in survival rates were observed. Our study supports the view that classical cytogenetic analysis remains an important tool regarding HSCT outcome.

## RESUMO

**CONTEXTO E OBJETIVO:**

Após o transplante de células tronco-hematopoéticas (TCTH), o cariótipo é uma ferramenta valiosa para monitorar o status do enxerto e da doença. Poucos estudos investigaram o significado prognóstico do cariótipo nos pacientes que se submeteram ao TCTH para leucemia mielóide crônica (LMC). O objetivo desse estudo foi verificar o significado dos achados citogenéticos pré-TCTH em pacientes portadores de LMC.

**TIPO DE ESTUDO E LOCAL:**

Série de casos. Instituto Nacional do Câncer (INCA), Rio de Janeiro, Brasil.

**METODOLOGIA:**

Foram realizados estudos citogenéticos por bandeamento G em 39 pacientes submetidos ao TCTH.

**RESULTADOS:**

Trinta e um pacientes estavam em fase crônica e oito em fase acelerada. Pré-TCTH, alterações cromossômicas adicionais ao cromossomo Philadelphia (Ph) foram observadas em 11 pacientes. A mais freqüente foi o duplo Ph observado em quatro casos. Após o TCTH, quimerismo total foi observado em 31 pacientes (79,5%). Desses, 23 (82,3%) apresentavam somente o cromossomo Ph. Quimerismo misto foi observado em sete pacientes, sendo três com alterações adicionais ao Ph. Um caso não apresentou resposta ao TCTH. Recaída citogenética associada com recaída clínica foi observada em cinco pacientes. Após o TCTH, 27 pacientes permanecem vivos e com remissão clínica e citogenética.

**CONCLUSÃO:**

Em nosso estudo a presença de alterações cromossômicas adicionais ao Ph, prévias ao TCTH, não foi associada com pior evolução, com risco de recaída, bem como não foi observada diferença entre as taxas de sobrevida. Nosso estudo sugere que a citogenética clássica permanece uma grande ferramenta no monitoramento do TCTH.

## INTRODUCTION

Chronic myeloid leukemia (CML) is a myeloproliferative disorder that is genetically characterized by the translocation t(9;22)(q34;q11), which results in a *BCR-ABL*gene fusion on the derivative chromosome 22, called the Philadelphia chromosome (Ph).^1,2^Additional cytogenetic abnormalities are generally considered to be an important step in the evolution of CML from the chronic phase (CP) to the terminal blast crisis (BC). Additional chromosomal changes are detected in 70-80% of BC cases^3^and in approximately 10% of Ph-positive CML in the CP at the time of the diagnosis.^4^Patients with karyotypic clonal evolution have generally been reported to have a worse clinical outcome.^5^

The current therapies include hemato-poietic stem cell transplantation (HSCT) and drug regimens like interferon alpha and imatinib mesylate. HSCT is associated with substantial morbidity and mortality and is limited to patients for whom a suitable donor is available.^6^ The results are better for patients who are allografted in the CP than in the accelerated phase (AP) or BC.^7^

Following HSCT, karyotyping is a valuable tool for monitoring engraftment and disease status. However, few studies have examined the prognostic significance of karyotyping findings among patients who underwent HSCT for CML.

## OBJECTIVE

The objective of this study was to evaluate the significance of pretransplantation cytogenetic status in relation to outcomes following HSCT.

## METHODS

### Type of study

This was a case series study.

### Patients

We analyzed cytogenetic data from 39 patients with CML who underwent HSCT from an identical sibling (n = 35) or from unrelated volunteer donors (n = 4) between January 2000 and May 2005. Of these patients, 31 (79.5%) were in the CP and eight (20.5%) were in the AP. The criteria for CP and AP were those of the International Bone Marrow Transplant Registry.^8^ There were 27 males and 12 females and their median age was 39 years (range: 17-57 years). All the patients received conditioning consisting of cyclophosphamide and busulphan. Graft-versus-host disease (GVHD) prophylaxis was provided by cyclosporin and methotrexate. The median observation period was 27 months (range: 6-48 months). This study was approved by the Ethics Review Committee of Instituto Nacional do Câncer (INCA) (Protocol 58/05).

### Cytogenetic studies

Chromosomal analysis on bone marrow cells was carried out before HSCT and one, three and six months subsequent to HSCT, and every six months thereafter, using standard G banding.^9^ The chromosomes were classified according to the International System for Human Cytogenetic Nomenclature (ISCN).^10^ At least 20 metaphases were analyzed per patient. The cytogenetic response after HSCT was defined according to the chimerism level: full chimeras (100% donor metaphases), mixed chimeras (% donor metaphases/% patient metaphases) and no response (100% patient metaphases).

### Statistical analysis and survival

The association between the cytogenetic status prior to HSCT and the cytogenetic response was analyzed using the χ^2^test. Survival rates were analyzed and survival curves were produced using the Kaplan-Meiermethod (SPSS software, SPSS Inc., Chicago, United States).

## RESULTS

### Cytogenetic findings prior to HSCT

Additional chromosomal abnormalities were found in 11 patients (28.2%): three patients in the CP (27.3%) and eight in the AP (72.7%). The most frequent additional abnormalities prior to HSCT were a double Ph, which was observed in four cases (36.4%), and trisomy 8 in two cases (18%). The other additional chromosomal abnormalities are described in Table 1. Patients 8 and 11 did not show the Ph chromosome, but were *bcr-abl*-positive according to the reverse transcription-polymerase chain reaction (RT-PCR).

**Table 1.** Characteristics and responses to hematopoietic stem cell transplantation (HSCT) among the enrolled patients who had additional chromosomal abnormalities

Case	Age/sex	Disease status at HSCT	Pre-HSCT karyotype	Chimerism level	Status
1	32/M	AP	47,XY,t(9;22)(q34;q11),+ der(22)t(9;22)(q34;q11)[5]/46,XY,t(9;22)(q34;q11)[20]	FC	Alive
2	42/M	AP	47,XY,+8,t(9;22)(q34;q11)[20]/46,XY,t(9;22)(q34;q11)[6]	FC	Dead
3	39/M	AP	48,XY,+8, t(9;22)(q34;q11),+der(22)t(9;22)(q34;q11)[7]//46,XY,t(9;22)(q34;q11)[11]	FC	Alive
4	40/M	AP	47,XY,t(9;22)(q34;q11),+ der(22)t(9;22)(q34;q11)[29]	MC	Dead
5	48/M	CP	46,XY,t(6;13)(q24;q13),t(9;22)(q34;q11)[24]	FC	Alive
6	41/M	AP	46,XY,t(9;22)(q34;q11),add(19)(q13)[25]	MC	Alive
7	30/M	CP	46,XY,t(9;22)(q34;q11),t(10;15)(p15;q22)[28]	FC	Alive
8	50/F	CP	46,XX,i(9)(q10),del(22)(q11)[20]	MC	Alive
9	56/M	AP	47,XY,t(9;22)(q34;q11),+ der(22)t(9;22)(q34;q11)[14]/46,XY,t(9;22)(q34;q11)[13]	FC	Alive
10	22/M	AP	46,XY,del(3)(q21),t(9;22)(q34;q11),i(17)(q10)[4]/46,XY,t(9;22)(q34;q11),i(17)(q10)[15]/46,XY,t(9;22)(q34;q11)[3]	FC	Alive
11	38/M	AP	46,XY,del(3)(p21),del(22)(q11)[32]	FC	Dead

AP = accelerated phase; CP = chronic phase; FC = full chimeras; MC = mixed chimeras; M = male; F = female.

### Cytogenetic studies following HSCT

Full chimerism was observed in 31 patients (79.5%). Among these, eight (25.81%) had presented additional abnormalities prior to HSCT. In these patients, karyotyping prior to HSCT showed one case of each of trisomy 8, t(6;13), t(10;15), del(3q), i(17q) and del(3p) and two cases of double Ph. Mixed chimerism was observed in seven patients, of whom three had had additional abnormalities: add(19p), i(9q)/del(22q) and double Ph. One case (2.5%) showed no response, and this case only showed the Ph chromosome prior to HSCT. The difference between the patients with a single Ph chromosome and the patients with additional abnormalities was not statistically significant (p = 0.51). In five patients (12.8%), cytogenetic relapse associated with clinical relapse after HSCT was observed, and two of these patients had had additional abnormalities: double Ph and add(19)(q13). The treatment administered in cases of relapse was transfusion of donor lymphocytes (DLI) and/or imatinib mesylate. Our results showed no differences between patients with the Ph chromosome and patients with additional chromosomal abnormalities in relation to relapse following HSCT (p = 0.91).

### Survival

**Figure 1.**
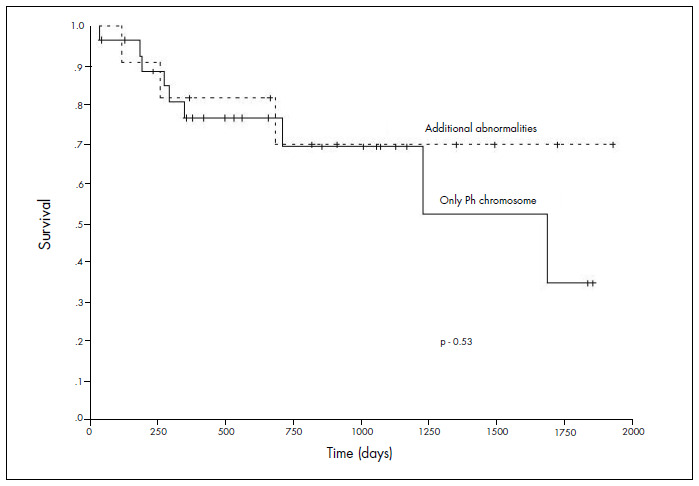
Overall survival according to karyotyping prior to hematopoietic stem cell transplantation (HSCT).

Twenty-seven patients are still alive and present complete hematological and cytogenetic remission. Eleven patients died from GVHD and/or severe infection and one patient died from relapse with disease evolution (lymphoid blast crisis) with the karyotype complex: 45,XY,dic(3;9)(q11; q11),del(5)(q31q35),-7,t(9;22)(q34;q11),-17,+2der(22)t(9;22)(q34;q11)[18]/5,XY,t(9;22)(q34;q11),dic(17;20)(q11;q11),-18,+der(22)t(9;22)(q34;q11)[4]. Among the patients who died, three (18.2%) had additional chromosomal abnormalities. The patients with the Ph translocation alone showed survival similar to that of patients with additional abnormalities (p = 0.53) (Figure 1).

## DISCUSSION

Additional chromosomal abnormalities prior to therapy such as interferon alpha or imatinib mesylate are associated with poor response and worse outcome.^11,12^ However, few studies have examined the effect of pre-HSCT cytogenetics on HSCT outcome. Przepiorka and Thomas^13^ examined 126 patients in the AP or BC and found additional cytogenetic abnormalities in 84% and variant Ph in 14%. The patients with variant Ph, and those with +8 or +Ph, showed a higher risk of relapse. Slovak et al.^14^ examined 21 patients in the AP and found that 10 showed additional cytogenetic abnormalities. No difference was found between those with and without additional abnormalities. Nevertheless, Konstantinidou et al.^15^ studied 418 patients in the pre-blastic phase who had undergone HSCT and observed that patients with standard Ph translocation, variant Ph translocation and negative for Ph may have different outcomes: Ph-negative patients showed a better outcome, and patients with variant Ph had a worse outcome than did the patients with standard Ph translocation. Patients with the additional changes of +8, +Ph and i(17q) do not necessarily show a worse outcome than do those with no additional changes, whereas those with other additional changes may fare worst of all. Although the number of cases was small, our results suggest that the presence of additional abnormalities was not associated with worse outcome and relapse risk, nor was it associated with any differences in survival rates.

## CONCLUSIONS

Our data suggest that patients with additional chromosomal abnormalities can be indicated for HSCT, since we did not observe any difference in cytogenetic response and survival rates between these patients and the patients only presenting a Ph chromosome.
